# Recognizing the Physiologic Signature of Oncologic Emergency: A Case of Burkitt Lymphoma Presenting With Early Laboratory Indicators of High Tumor Burden

**DOI:** 10.7759/cureus.107169

**Published:** 2026-04-16

**Authors:** Alireza Izadian Bidgoli, Alberto Gomez Veliz

**Affiliations:** 1 Internal Medicine, American University of the Caribbean School of Medicine, Cupecoy, SXM; 2 Internal Medicine, Jackson Memorial Hospital, Miami, USA

**Keywords:** burkitt lymphoma, diagnostic delay, lactate dehydrogenase, oncologic emergency, tumor burden

## Abstract

Burkitt lymphoma is a highly aggressive B-cell malignancy characterized by rapid proliferation and a narrow therapeutic window in which early recognition of disease progression is critical to prevent morbidity. While diagnosis is typically established through histopathology and imaging, evolving physiologic and radiographic changes may provide early indicators of aggressive disease behavior, particularly in the post treatment setting. We present a 26-year-old male patient with previously treated Epstein-Barr virus-positive Burkitt lymphoma who presented with acute lower back pain, urinary symptoms, and rapid clinical deterioration consistent with disease progression. Laboratory evaluation demonstrated normocytic anemia, markedly elevated lactate dehydrogenase, progressive leukocytosis, and hypoalbuminemia, consistent with a high tumor burden. Cross-sectional imaging revealed interval enlargement of a necrotic pelvic mass with associated hydronephrosis, while MRI demonstrated diffuse marrow infiltration. Fluorodeoxyglucose-Positron emission tomography/Computed tomography (FDG PET/CT) showed extensive hypermetabolic disease involving nodal and extranodal sites, consistent with systemic progression. Peripheral blood flow cytometry failed to identify a clonal B-cell population, a finding consistent with known limitations of circulating markers in Burkitt lymphoma, particularly following recent chemotherapy. This case highlights a physiologic pattern of oncologic emergency, defined by integrated laboratory abnormalities, imaging progression, and clinical decline as indicators of disease progression. Recognition of these features may facilitate timely therapeutic escalation despite inconclusive peripheral findings.

## Introduction

Burkitt lymphoma is a highly aggressive mature B-cell neoplasm driven by MYC rearrangement, characterized by rapid proliferation and one of the shortest doubling times among human malignancies [1]. This biologic behavior results in abrupt disease progression, high tumor burden, and a narrow therapeutic window in which early recognition is critical to prevent life-threatening complications [1,2]. In adults, the disease commonly presents with bulky extranodal involvement, particularly within the abdomen, and may progress rapidly even over short clinical intervals [2-4].

Although diagnosis is traditionally established through histopathologic confirmation and imaging, emerging evidence suggests that physiologic and laboratory abnormalities may precede definitive diagnostic findings, reflecting the underlying metabolic intensity of the disease [2-4]. Elevated lactate dehydrogenase (LDH) is a well-established surrogate marker of tumor burden and cellular turnover in aggressive lymphomas and has been incorporated into prognostic models to stratify disease severity [4,5]. Additional abnormalities, including anemia and evolving leukocytosis, may further signal an underlying high-grade malignancy but are often non-specific and can delay recognition when interpreted in isolation [1-3].

The clinical challenge lies in recognizing these findings as part of a broader physiologic pattern of oncologic emergency [1,2]. In Burkitt lymphoma, disease progression is exponential rather than linear, and delays in diagnosis are biologically amplified, increasing the risk of complications such as tumor lysis syndrome and rapid clinical decline [1,2,4]. Furthermore, extensive disease burden may exist in the absence of circulating malignant cells, underscoring the limitations of peripheral diagnostic markers in accurately reflecting disease activity, particularly in the post treatment setting [3,4].

We report a case of previously treated Burkitt lymphoma in which evolving laboratory abnormalities, rapid clinical deterioration, and advanced imaging findings signaled aggressive disease progression despite non-diagnostic peripheral flow cytometry. This case highlights a critical limitation of conventional peripheral diagnostic markers and emphasizes the importance of integrating physiologic and radiographic data in assessing disease activity [3,4]. We propose that, in aggressive lymphomas such as Burkitt lymphoma, early recognition of a high tumor burden should rely on dynamic clinical, laboratory, and imaging patterns rather than isolated diagnostic tests, which may support earlier identification of disease progression and timely therapeutic escalation even in the absence of definitive peripheral findings [1-4].

## Case presentation

Clinical presentation 

A 26-year-old male patient with a past medical history of Epstein-Barr virus (EBV)-positive Burkitt lymphoma, diagnosed in January 2026, presented with acute-onset, progressively worsening lower back pain radiating to the right thigh. The pain began two days prior to admission and rapidly escalated in severity, becoming intolerable on presentation. He additionally reported urinary hesitancy with straining and decreased urinary output. He denied fever, chills, nausea, vomiting, or abdominal pain.

His recent clinical course was notable for completion of systemic chemotherapy for Burkitt lymphoma; however, the exact chemotherapy regimen, including whether rituximab was administered, was not available in the medical records. His course was further complicated by infected tumor necrosis and tumor-enteric fistula formation, requiring intensive care unit admission, interventional radiology-guided drainage, and broad-spectrum antimicrobial therapy. Although initially deemed a poor surgical candidate due to profound neutropenia and septic shock, he subsequently underwent exploratory laparotomy with adhesiolysis and resection of a right pelvic mass.

At the time of presentation, he also noted the recent development of a new lesion within the left inguinoscrotal region.

Physical exam and initial assessment

On examination, the patient was alert and oriented, appearing chronically ill but in no acute distress. Vital signs were notable for tachycardia, with a heart rate of 123 beats per minute (reference range: 60-100 beats per minute); all other parameters were hemodynamically stable, and oxygen saturation was normal on room air. Abdominal examination demonstrated a soft, non-distended abdomen with a well approximated midline surgical incision without signs of infection. A poorly defined area of firmness was palpated in the right lower quadrant, without associated tenderness.

**Figure 1:**
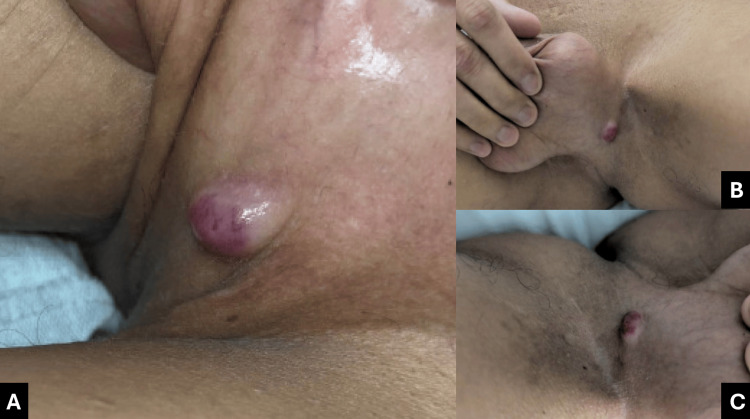
Lesion within the left inguinoscrotal crease (A) Close-up view demonstrating a violaceous, round nodular lesion within the left inguinoscrotal crease; (B) Intermediate view illustrating the lesion in relation to the surrounding inguinal and scrotal anatomy; (C) Wider field view demonstrating the overall distribution and localization of the lesion within the left inguinoscrotal region.

Genitourinary examination demonstrated left costovertebral angle tenderness. A distinct, non-tender, violaceous, round nodular lesion was identified within the left inguinoscrotal crease (Figure 1).

Neurologic examination was grossly intact, although the patient reported radicular pain extending into the right lower extremity. The remainder of the examination was unremarkable.

Laboratory evaluation revealed leukocytosis, chronic anemia, and markedly elevated LDH levels, consistent with high tumor burden. Renal function remained preserved despite clinical suspicion for evolving obstructive uropathy.

Laboratory results and specialized studies

Initial laboratory evaluation revealed normocytic anemia and markedly elevated lactate dehydrogenase (LDH), consistent with high cellular turnover. Serial studies demonstrated progressive leukocytosis, persistent anemia, and hypoalbuminemia, collectively reflecting increasing tumor burden. Despite these findings, there was no clinical or laboratory evidence of tumor lysis syndrome. Renal function, uric acid, potassium, and calcium levels remained within normal limits throughout hospitalization, with no features suggestive of spontaneous or treatment-related tumor lysis. Renal function also remained preserved despite clinical concern for obstructive uropathy. A comprehensive summary of laboratory findings is provided in Table 1.

**Table 1:** Laboratory findings at admission and during hospitalization Laboratory values at admission and the most clinically significant values recorded during hospitalization are presented. Findings are notable for normocytic anemia, progressive leukocytosis, elevated lactate dehydrogenase levels consistent with high cellular turnover, and hypoalbuminemia. Renal function remained preserved throughout the hospital course. Reference ranges and units are provided for all parameters. AST: aspartate aminotransferase; ALT: alanine aminotransferase; SGCOT: serum glutamic oxaloacetic transaminase; SGPT: serum glutamic pyruvate transaminase.

Laboratory test	At admission	During hospitalization (Range)	Reference range (Units)
Hemoglobin (g/dL)	8.1	7.3–8.5	13.5–17.5
White blood cell count (×10³/µL)	4.9	12.1–17.3	4.0–11.0
Platelet count (×10³/µL)	426	315–426	150–400
Lactate dehydrogenase (LDH; U/L)	918	703–918	140–280
Creatinine (mg/dL)	0.50	0.47–0.60	0.6–1.3
Blood Urea Nitrogen (BUN; mg/dL)	8	4–10	7–20
Sodium (mmol/L)	137	135–138	135–145
Potassium (mmol/L)	4.6	3.5–4.6	3.5–5.0
Calcium (mg/dL)	8.6	8.2–8.8	8.5–10.5
Total protein (g/dL)	5.6	5.2–5.9	6.0–8.3
Albumin (g/dL)	3.0	2.7–3.1	3.5–5.0
AST (SGOT; U/L)	50	29–50	10–40
ALT (SGPT; U/L))	21	20–26	7–56
Alkaline phosphatase (U/L)	49	51–87	44–147
Uric acid (mg/dL)	3.5	3.1–4.1	3.5–7.2

**Figure 2:**
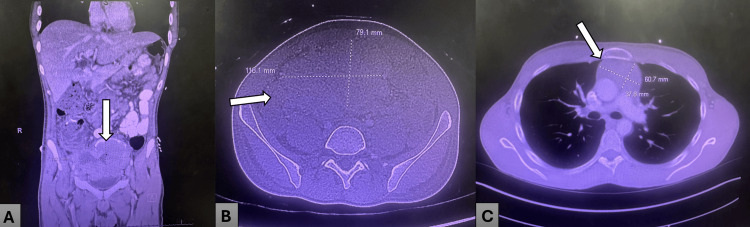
Cross-sectional imaging demonstrating bulky pelvic mass and disease burden. (A) Coronal CT image demonstrating a large pelvic mass centered superior to the bladder (arrow), consistent with bulky extranodal lymphoma involvement; (B) Axial CT image of the pelvis demonstrating the mass with maximal dimensions measuring approximately 11.8 × 7.9 cm (arrow), reflecting significant tumor burden; (C) Axial CT image of the chest demonstrating additional thoracic involvement with enlarged mediastinal lymph nodes (arrow), supporting disseminated disease.

Contrast-enhanced computed tomography (CECT) of the abdomen and pelvis demonstrated interval enlargement of a large, centrally necrotic pelvic mass measuring approximately 11.8 cm, with associated bulky retroperitoneal lymphadenopathy and significant mass effect resulting in left-sided hydronephrosis and delayed nephrogram (Figure 2).

The lesion demonstrated features of aggressive malignancy, including central necrosis and heterogeneous architecture.

Magnetic resonance imaging (MRI) of the spine demonstrated diffuse marrow signal abnormalities throughout the axial skeleton, consistent with widespread marrow infiltration. No evidence of epidural extension, spinal canal compromise, or cord compression was identified. MRI of the brain revealed no intracranial involvement. These findings further supported systemic dissemination in the absence of focal neurologic compromise.

**Figure 3:**
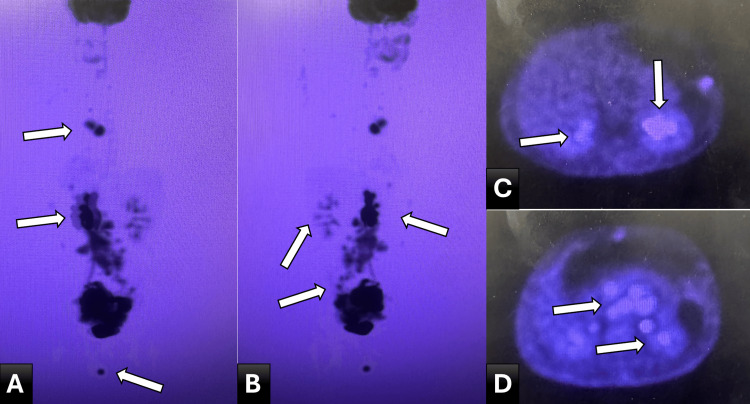
FDG PET/CT maximum intensity projection (MIP) images demonstrating extensive systemic disease. (A, B) Whole-body Fluorodeoxyglucose-Positron Emission Tomography/Computed Tomography (FDG PET/CT) MIP images demonstrating intense hypermetabolic activity involving a dominant pelvic mass with widespread nodal and extranodal uptake, consistent with disseminated high-grade lymphoma. The distribution and intensity of uptake reflect a high tumor burden; (C) Axial PET/CT image demonstrating focal hypermetabolic activity corresponding to extranodal involvement, including the inguinoscrotal region. (D) Axial PET/CT image demonstrating heterogeneous metabolic activity within the pelvic mass, consistent with aggressive tumor biology and areas of necrosis.

Functional imaging with fluorodeoxyglucose positron emission tomography/computed tomography (FDG PET/CT) demonstrated markedly hypermetabolic disease (Standard uptake value (SUV) >10) involving the dominant pelvic mass, mediastinal lymph nodes, and multiple nodal and extranodal sites (Figure 3).

Whole-body maximum intensity projection images revealed extensive metabolic activity consistent with a high tumor burden. Axial PET/CT images further demonstrated heterogeneous uptake within the pelvic mass, corresponding to areas of viable tumor interspersed with necrosis. Additional foci of hypermetabolism, including involvement of the inguinoscrotal region, reinforced the presence of progressive systemic dissemination.

Duplex ultrasonography of the bilateral lower extremities demonstrated normal venous compressibility and flow, without evidence of deep venous thrombosis.

Tissue diagnosis

Histopathologic evaluation of the previously resected pelvic mass confirmed Burkitt lymphoma, a high-grade B-cell neoplasm with features consistent with EBV-associated disease. The patient recently completed systemic chemotherapy for Burkitt lymphoma prior to this admission; however, the exact regimen was not specified in the available records.

During the current hospitalization, peripheral blood flow cytometry performed using a B-cell lymphoma panel, including markers such as cluster of differentiation (CD)19, CD20, CD10, CD5, CD23, and light chains, did not demonstrate an immunophenotypically abnormal B-cell population. CD19-positive B cells were essentially absent, a finding that may reflect prior exposure to B-cell depleting therapy, although this cannot be confirmed given the lack of documented chemotherapy details. In the context of recent chemotherapy, these findings are most consistent with treatment effect and are insufficient to exclude persistent or progressive lymphoma, particularly given the presence of known bulky disease on imaging.

Although biopsy of the cutaneous lesion was not performed during this hospitalization, its clinical appearance and temporal association with radiographic progression, including interval enlargement of a necrotic pelvic mass and worsening retroperitoneal lymphadenopathy, raise concern for possible cutaneous involvement by lymphoma, although this remains unconfirmed.

Multidisciplinary tumor board discussion

The case was reviewed in a multidisciplinary conference involving medical oncology, surgical oncology, radiology, and pathology. The patient’s clinical deterioration, imaging findings, and rising tumor burden were collectively interpreted as evidence of aggressive disease progression despite recent therapy.

Notably, the absence of circulating malignant B-cells on peripheral flow cytometry was considered discordant with the extensive intra-abdominal disease burden, highlighting the limited sensitivity of peripheral assays in accurately reflecting disease activity in aggressive lymphomas.

Given the patient’s recent postoperative state, prior infectious complications, and overall clinical fragility, he was not considered a candidate for repeat surgical intervention. The consensus favored continuation and potential escalation of systemic therapy, with close clinical and radiographic monitoring.

The inguinoscrotal lesion was deemed highly suspicious for extranodal lymphoma involvement in the context of systemic progression.

Treatment

The patient was managed with continued systemic chemotherapy under the direction of the oncology team. He had previously received systemic chemotherapy for Burkitt lymphoma prior to this admission; however, the specific regimen and inclusion of rituximab were not clearly documented in the available medical records.

During the current hospitalization, management focused on treatment of progressive disease in conjunction with supportive care measures, including analgesia and close monitoring of renal function in the setting of hydronephrosis. Given the patient’s recent postoperative state, prior infectious complications, and overall clinical status, therapeutic decisions were guided by a multidisciplinary oncology team with ongoing reassessment of treatment response.

Clinical outcome 

During hospitalization, the patient remained hemodynamically stable while undergoing continued inpatient systemic chemotherapy. He has tolerated treatment well without significant acute complications. His clinical course has been notable for gradual improvement in symptom burden, particularly with respect to pain control.

The patient previously underwent successful surgical resection of the intra-abdominal tumor, and the surgical site has demonstrated appropriate healing without evidence of infection or dehiscence. Ongoing multidisciplinary management has focused on continued oncologic therapy, supportive care, and close monitoring for treatment response and potential complications.

At the time of this report, the patient remains hospitalized, actively receiving chemotherapy with close inpatient follow-up.

## Discussion

Background (history, epidemiology, and WHO classification)

Burkitt lymphoma is a highly aggressive mature B-cell non-Hodgkin lymphoma first described by Denis Burkitt in 1958 in equatorial Africa, where it was initially recognized as a rapidly growing pediatric jaw tumor with a distinctive geographic distribution [1]. It is now understood as a biologically defined lymphoma driven by MYC rearrangement and classified into endemic, sporadic, and immunodeficiency associated variants, each with distinct epidemiologic and clinical characteristics [1,2].

In adults, Burkitt lymphoma is rare, accounting for less than 5% of adult lymphomas, although it occurs across the age spectrum and is not limited to pediatric populations [2,5]. Sporadic Burkitt lymphoma, the form most commonly encountered in Western countries, frequently presents with abdominal extranodal disease, particularly involving the gastrointestinal tract [2,5,6].

Risk factors vary by subtype. Endemic Burkitt lymphoma is strongly associated with EBV infection and Plasmodium falciparum malaria, whereas immunodeficiency associated Burkitt lymphoma occurs in the setting of HIV infection and other states of immune suppression [7,8]. Despite these associations, many sporadic cases arise without a clearly identifiable precipitating factor [2,8].

Tumor biology is characterized by an exceptionally high proliferative rate, with Ki-67 approaching 100%, reflecting rapid tumor doubling and aggressive clinical behavior [2,6]. Burkitt lymphoma commonly involves extranodal sites, including the gastrointestinal tract, bone marrow, and central nervous system, depending on disease subtype and stage [2,6]. According to the 5th edition of the World Health Organization (WHO) classification of haematolymphoid tumors, Burkitt lymphoma is categorized as a high grade B-cell neoplasm defined by MYC rearrangement and a characteristic immunophenotypic profile [1]. Despite its aggressive nature, it is highly responsive to intensive chemotherapy, and outcomes are strongly dependent on timely diagnosis and treatment initiation [2,6].

Pathogenesis and pathophysiology

Burkitt lymphoma is driven by translocation induced dysregulation of the MYC oncogene, most commonly t(8;14), resulting in constitutive cellular proliferation, metabolic upregulation, and impaired apoptosis [1,4]. This molecular hallmark produces one of the highest proliferative indices among human malignancies, with Ki-67 approaching 100%, accounting for the tumor’s rapid doubling time and aggressive clinical behavior [1,2].

The resulting high cellular turnover generates a hypermetabolic state characterized by elevated LDH, a surrogate marker of tumor burden and cellular turnover [9]. This metabolic intensity underlies the propensity for early metabolic complications, including tumor lysis physiology, which may occur even prior to therapy initiation [2,4].

EBV, particularly in endemic and a subset of sporadic cases, contributes to lymphomagenesis through latent gene expression that promotes B-cell survival and proliferation, further augmenting MYC driven oncogenesis [1,10].

Importantly, disease burden is often compartmentalized, and peripheral blood findings may underestimate systemic involvement [4,11,12]. This results in potential discordance between circulating markers and true disease activity, as demonstrated in this case, reinforcing that Burkitt lymphoma behaves as a rapidly evolving, systemically active malignancy defined by its underlying biology rather than static diagnostic measures [4,11,12].

Comparative analysis with the existing literature

Clinical Presentation

Adult Burkitt lymphoma is a highly aggressive malignancy characterized by rapid tumor growth and a high proliferative index, often presenting with bulky extranodal disease, particularly within the abdomen [2,5,6]. Patients commonly develop symptoms related to mass effect, including abdominal or back pain, obstructive uropathy, and functional decline, frequently accompanied by elevated LDH as a marker of tumor burden and cellular turnover [2-4,6].

In this case, the patient presented with acute back pain, urinary symptoms, and rapid clinical deterioration in the setting of previously treated Burkitt lymphoma, consistent with the known aggressive clinical behavior of the disease [2,4]. Imaging demonstrated a large necrotic pelvic mass with associated hydronephrosis and progressive lymphadenopathy, reflecting advanced systemic involvement. While intra-abdominal disease is well described in Burkitt lymphoma, this case illustrates how extensive tumor burden may lead to complex clinical presentations with overlapping oncologic and systemic complications.

Diagnostic Workup

The diagnosis of Burkitt lymphoma is established through tissue biopsy with immunophenotypic and molecular characterization, including MYC rearrangement, and is supported by imaging for staging and disease assessment [1,2,6]. Laboratory abnormalities, particularly elevated LDH, are commonly used as surrogate markers of tumor burden and are associated with aggressive disease [4,5].

In this patient, laboratory findings demonstrated markedly elevated LDH (peaking at 918 U/L), anemia, and evolving leukocytosis, consistent with a high tumor burden [2-4,6]. Imaging revealed interval enlargement of a necrotic pelvic mass, extensive lymphadenopathy, and diffuse marrow infiltration, supporting widespread disease progression [2,6].

A notable feature of this case was the discordance between peripheral blood findings and systemic disease burden. Peripheral blood flow cytometry did not demonstrate a clonal B-cell population despite extensive radiographic disease. While Burkitt lymphoma may involve the peripheral blood, circulating malignant cells are not uniformly present, and peripheral studies may not fully reflect disease extent, particularly in the setting of compartmentalized disease or following treatment [4,6]. This highlights the importance of integrating clinical, laboratory, and imaging findings when evaluating disease activity in aggressive lymphomas [3,4].

Management

Burkitt lymphoma is primarily treated with intensive multi agent chemotherapy regimens, often including rituximab, and outcomes are closely linked to early initiation of therapy [2,6]. Surgical intervention is not a standard component of treatment but may be required in selected cases involving complications of bulky abdominal disease [2,6].

In this case, management was complicated by progressive lymphoma in the setting of severe intra-abdominal pathology, including a necrotic tumor mass and associated clinical instability. The patient underwent surgical resection of the pelvic mass and involved bowel as part of management of these complications. This approach reflects an individualized, multidisciplinary decision making process in which surgical intervention was necessary to address acute clinical issues and allow continuation of oncologic therapy.

Such cases underscore that, although chemotherapy remains the cornerstone of Burkitt lymphoma treatment, management strategies may need to be adapted in the presence of complications related to tumor burden or treatment effects, requiring close coordination between oncology, surgery, and supportive care teams [2,6].

Outcome

Despite significant disease burden, the patient remained hemodynamically stable during hospitalization and was able to continue systemic therapy. His clinical course demonstrated improvement in symptom burden, particularly pain control, although laboratory and imaging findings indicated ongoing disease activity.

This outcome reflects the known chemosensitivity of Burkitt lymphoma despite its aggressive biology and supports the importance of maintaining continuity of therapy when clinically feasible [2,6]. It also highlights the role of supportive and multidisciplinary care in stabilizing patients with complex disease presentations.

Literature Summary

This case is consistent with established literature describing Burkitt lymphoma as a rapidly progressive malignancy with high tumor burden and frequent extranodal involvement [2-4,6]. It further reinforces several important clinical principles.

First, laboratory abnormalities such as elevated LDH, in conjunction with imaging findings, provide important insight into disease activity and tumor burden [4,5]. Second, peripheral diagnostic studies may not fully reflect systemic disease extent, emphasizing the need for integrated clinical assessment [4,6].

Finally, while Burkitt lymphoma is primarily managed with chemotherapy, this case illustrates that clinical management may require individualized approaches in the presence of complications, highlighting the importance of multidisciplinary care. Overall, these findings support a comprehensive, biology driven approach to evaluating and managing aggressive lymphomas, integrating clinical, laboratory, and imaging data to guide timely and appropriate intervention [3,4].

Learnings

This case highlights that in Burkitt lymphoma, disease progression may be clinically and biologically apparent despite non-diagnostic peripheral studies, particularly in the post treatment setting. In this patient, marked elevations in LDH, evolving cytopenias, and rapid radiographic progression reflected a high tumor burden, while peripheral blood flow cytometry remained negative. This discordance underscores a key limitation of relying on circulating markers to assess disease activity in aggressive lymphomas.

Importantly, this case also illustrates the potential for Burkitt lymphoma to present with overlapping oncologic and surgical emergencies. The development of tumor necrosis, fistulization, and sepsis necessitated surgical intervention, emphasizing that management may require deviation from standard chemotherapy driven approaches in the setting of life threatening complications.

Together, these findings support a biology driven approach to clinical decision making, in which dynamic laboratory trends, imaging findings, and clinical trajectory are prioritized over isolated diagnostic tests. Early recognition of these patterns may facilitate timely intervention, including escalation of therapy or multidisciplinary management, even when conventional diagnostic markers are inconclusive.

A key limitation of this case is the absence of detailed documentation regarding the patient’s prior chemotherapy regimen, including whether rituximab was administered. This restricts precise interpretation of treatment response and limits the ability to distinguish between refractory disease and early relapse following standard therapy. Additionally, prior exposure to B-cell-depleting agents, such as rituximab, may partially explain the absence of circulating CD19-positive B cells on peripheral flow cytometry, further complicating interpretation of disease activity. Despite this limitation, the patient’s clinical deterioration, laboratory abnormalities, and imaging findings consistently supported progressive high tumor burden.

## Conclusions

Burkitt lymphoma is a malignancy in which timely recognition depends on the ability to translate early clinical, laboratory, and radiographic findings into meaningful clinical action. This case underscores that abnormalities such as elevated lactate dehydrogenase, progressive cytopenias, and rapidly evolving imaging findings should prompt early suspicion for high tumor burden and aggressive disease, even in the absence of definitive peripheral diagnostic confirmation. Accordingly, effective management requires early multidisciplinary integration and a low threshold for therapeutic escalation in the setting of concerning physiologic patterns. In clinical practice, this approach may support earlier use of advanced imaging and broader interpretation of laboratory abnormalities as indicators of disease progression, particularly when peripheral diagnostic studies are inconclusive. Recognizing these early indicators as clinically actionable may reduce diagnostic delay and improve outcomes in rapidly progressive malignancies such as Burkitt lymphoma.
